# “Let me know when I’m needed”: Exploring the gendered nature of digital technology use for health information seeking during the transition to parenting

**DOI:** 10.1177/20552076211048638

**Published:** 2021-10-18

**Authors:** Bradley Hiebert, Jodi Hall, Lorie Donelle, Danica Facca, Kim Jackson, Ewelina Stoyanovich

**Affiliations:** 1 Arthur Labatt Family School of Nursing, Western University, London, Canada; 2 School of Nursing, 113612 Fanshawe College, London, Canada; 3 Faculty of Information and Media Studies, 70383 Western University, London, Canada

**Keywords:** Digital health, technology, health information seeking, gender, reproductive health, sexual health, connected care, gender

## Abstract

This paper presents results of a qualitative descriptive study conducted to understand parents’ experiences with digital technologies during their transition to parenting (i.e. the period from pre-conception through postpartum). Individuals in southwest Ontario who had become a new parent within the previous 24 months were recruited to participate in a focus group or individual interview. Participants were asked to describe the type of technologies they/their partner used during their transition to parenthood, and how such technologies were used to support their own and their family's health. Focus group and interview transcripts were then subjected to thematic analysis using inductive coding. Ten focus groups and three individual interviews were conducted with 26 heterosexual female participants. Participants primarily used digital technologies to: (1) seek health information for a variety of reproductive health issues, and (2) establish social and emotional connections. The nature of such health information work was markedly gendered and was categorized by 2 dominant themes. First, “‘Let me know when I’m needed’”, characterizes fathers’ apparent avoidance of health information seeking and resultant creation of mothers as lay information mediaries. Second, “Information Curation”, captures participants’ belief that gender biases built-in to popular parenting apps and resources reified the gendered nature of health and health information work during the transition to parenting. Overall, findings indicate that digital technology tailored to new and expecting parents actively reinforced gender norms regarding health information seeking, which creates undue burden on new mothers to become the sole health information seeker and interpreter for their family.

Health information seeking is a goal-oriented process used by an individual to address concerns or uncertainties they have about a health-related issue.^1,2^ Information source is a fundamental element of health information seeking as different sources will support an individual's information needs in different contexts.^3,4^ During the transition to parenting, that is the period from pre-conception through postpartum and the first 2 years of childrearing, individuals and families have significant health information needs. Consequently, they employ diverse health information seeking strategies from a variety of sources.^5,6^

The rapid proliferation of digital technologies – understood as any computer-dependant device and all applications that run on them – has ostensibly provided many new and expecting parents the ability to meet their health information needs more rapidly and conveniently than ever before.^5,7^ During the transition to parenting mothers and fathers share common health information needs, including fetal development, stages of pregnancy, and breastfeeding,^8,9^ however there are disparities in the breadth of health information sources mothers and fathers consult, and their motivations.

Mothers and fathers frequently cite the Internet as a source of health information commonly consulted during the transition to parenting, however, they often report being overwhelmed by the amount of information available online.^7,10^ To overcome anxiousness associated with seeking health information online, new and expecting parents often consult individuals with expert or tacit knowledge of their health concern. For example, new and expecting mothers tend to actively seek health information from a variety of formal and informal sources, including physicians, midwives, family, and friends as their most trusted sources for health information related to the transition to parenting.^7,9^ Conversely, new and expectant fathers tend to be reluctant to search for health information and often cite their female partners as their single most trusted health information source during this period.^9,11^

The divergence in health information seeking approaches of mothers and fathers during the transition to parenting is influenced by historical sociocultural gendered divisions of labour.^12^ For example, awareness and recognition of health issues is often perceived as feminine behaviour.^12^ This may limit how readily new and expectant fathers look for health information during the transition to parenting, as doing so may challenge their embodiment of Westernized constructs of masculinity.^2^ Such gender norms create undue health work for new and expecting mothers who are required to adopt the role of lay information mediary and conduct most of, if not all, of the health information seeking work for their families.^13^ Now, this role of lay information mediary extends itself into online spaces.

Despite a growing body of literature on the health information preferences of new and expecting mothers and fathers,^7,9,11,14^ there is limited knowledge about the possibilities for how the gendered nature of health information seeking during the transition to parenting (re)configures the uses of digital technologies. Understanding how digital technologies are used during their transition to parenting would allow health service providers to tailor health information delivery methods to better meet the needs of new parents. Drawing on findings from a larger study which aimed to understand how digital technologies influenced parenting practices during the transition to parenting,^10^ this paper specifically examines how such technologies were used according to gendered norms among new and expectant parents seeking health information during this transition.

## Methods

This qualitative descriptive study^15^ was conducted to understand new parents’ experiences with, and uses of digital technology, during four stages of their transition to parenting: prenatal, pregnancy, labour and delivery, and postpartum. The research team was interdisciplinary, with representation from nursing, health studies, health professional education, public health, and doula studies. Most members of the team were parents, with children of various ages, and had their own varied experiences with using digital technologies to support health information seeking in their roles as parents.

### Recruitment

This study took place during 2018–2019 in an urban setting in southwest Ontario, Canada. A purposive sampling strategy was implemented using snowball sampling techniques^16^ to recruit individuals who were expectant parents or who had become a parent within the previous 24 months to participate in a focus group. Recruitment flyers were posted in locations where new parents were believed to frequent, such as local public health units, daycare centres, family health clinics, and early years play centres. Digital flyers and advertisements were also purchased on online buy-and-sell websites, and social media platforms such as Facebook. All recruitment materials were gender inclusive to recognize not all people who become pregnant and a parent fit the categories “woman”/“mother” or “man”/“father”. Interested individuals were eligible to participate if they met the following inclusion criteria: (1) identified as an expectant or new parent who had recently undergone the transition to parenting within the last 24 months; (2) were between 16 and 35 years old; and (3) were fluent English speaking. All participants provided written informed consent prior to participating in this study and were provided with a $15 honorarium immediately after providing consent and before engaging in any other research activities.

### Data collection & analysis

Focus groups were conducted by members of the research team in locations agreed upon between participants and researchers, including public libraries, a youth shelter, and a children's centre. The nature of inquiry within the focus groups was related to participants’ use of digital technologies. For example, participants were asked to describe the type of technologies they/their partner used during their transition to parenting, and how such technologies were used to support their own and their family's health. If a focus group participant introduced a topic that required additional time or consideration to discuss, an individual follow-up interview was offered. A demographic questionnaire was given to each participant at the outset of the focus group to elicit descriptive characteristics of the participants. Data were digitally recorded and transcribed verbatim concurrently with thematic data analysis. Field notes by researchers were also utilized to document relevant data not able to be captured by the digital recording, such as non-verbal communication.

Recruitment occurred concurrently with data analysis and continued until data saturation was met and no new themes, patterns, or ideas were generated by participants.^15,17^ An iterative thematic analysis approach^18^ was used by the research team to co-construct the study findings as each research team member independently analyzed every transcript and recorded their own analytic thoughts and thematic codes to describe the data. Individual analyses were then compared and discussed via in-person dialogue during team meetings, and the themes and codes that emerged from these discussions were recorded in a matrix alongside transcript quotes that best reflected each concept.

## Findings

### Participant characteristics

Ten focus groups (consisting of two to four participants per group) and three one-on-one follow-up semi-structured interviews were conducted with 26 participants. Despite recruiting ‘new parents’ with a broad set of inclusion criteria and no limitations to gender or sexual orientation, all participants identified as cis-gendered, heterosexual women. Participants ranged from 17 to 35 years old (eight were <20 years old, four were between 21 and 29 years old, and 10 were between 30 and 35 years old), and reflected a broad range in education (at the time of recruitment seven were completing secondary school, one had completed high school, one had completed community college, 10 had completed a university undergraduate degree, and two had completed a graduate degree), employment status (nine were unemployed, three reported part-time employment, seven reported full-time employment, and six did not share their employment status), and annual household income in Canadian dollars (four reported annual household income of <$20,000, three reported between $20,000 and $49,999, four reported between $50,000 and $99,999, and five reported $100,000 or greater). Half of the participants indicated that they were married, seven indicated that they were single and had never been married, and one was separated from her partner. Majority of participants (*N* = 18) identified as Caucasian, three identified as a racialized group, and five did not disclose their race.

Participants used a variety of digital technologies during their transition to parenting, including a range of devices (e.g. smartphones, tablets, laptops, TVs, baby monitors, and Dopplers), online resources (e.g. text messaging, video streaming services, social media sites such as Facebook or Snapchat, and search engines), and apps (e.g. Period Tracker, What to Expect, Bump, O Mama, Safety First, and Baby Tracker); for details regarding the type of devices used see Donelle et al.^10^ Participants primarily used digital technologies to (1) seek health information for a variety of issues including ovulation tracking, fetal development, infant feeding, infant health and developmental milestones, and maternal health at different stages along the transition to parenting – and (2) establish and maintain social and emotional connections. As participants described, the nature of such health information work was markedly gendered and was categorized by two dominant themes. First, “‘Let me know when I’m needed’”, characterizes how digital technologies were used for health information work among participants while highlighting the apparent avoidance of health information work among fathers, and the resultant creation of mothers as digital lay information mediaries. Second, “Information curation”, captures participants’ belief that gendered biases inherent to popular parenting apps and online information resources reified the gendered nature of health information work more broadly during the transition to parenting.

### “Let me know when I’m needed”: gendered nature of health information work

All participants – regardless of their age, education, income, marital status, or pregnancy history – offered similar descriptions of how their own and their male partners’ digital technology use for health information work appeared to be divided based on gender. Based on participants’ experiences, such differences in digital technology use were conceptualized by their own and how they perceived their male partners’: (1) direct use of digital technology for health information work, and (2) attitudes regarding the use of digital technology for health information work.

### Direct use of digital technologies for health information work

Participants all shared the perspective that the direct use of digital technologies for health information work and other health-related purposes was their responsibility, while their partners sought to actively absolve themselves from such activities. For example, one participant described the instant when her partner overtly removed himself from the digital health information work: “No that was up to you [referring to herself as the mother] … he said, ‘Let me know when I’m needed.’” Participants often described how this sentiment from their partners manifested in their digital technology use, with one participant providing a clear example of how this influenced the gendered division of health information work between her and her partner:

But like all of his gaming buddies and stuff know about her and have seen pictures and those kinds of things. I’m usually the one that's Googling if there's any question of whether a concern- or whether we should take her to the doctor or those kinds of things. I’m usually the one that does that. [T2]

In response to their partners’ explicit self-removal from digital health information work, these participants described how they would look for and share specific health information resources with their partners to promote their engagement with the pregnancy and subsequent decision making via the digital material. However, some participants acknowledged this method of health information sharing rarely guaranteed their partner would engage with the information they shared. One mother described this experience with frustration when she said, “I sent him lots of articles and they just got lost in his inbox.”

Participants described occasional instances when their partners did engage in health information work using digital technologies. During these examples participants highlighted their partners’ tendencies to hide that they were using digital technologies for health information seeking until the participants confronted them about it. One mother described an experience that resonated with others in her focus group:

I feel like my husband will, like sometimes, like he’ll hide it from me that he's looking something up because he knows I’m so anxious and Googling things and if he's actually concerned about something, I know he will look at like- start Googling it too. But then it won’t be until like I look on his phone that I know he's looked something up. [T11]

Participants also overwhelmingly shared the experience of being their partners’ apparent first choice for health information related to the transition to parenting, especially when participants were away from their child(ren). Multiple participants suggested that the ubiquity of digital technologies enabled their partners to hold participants in their role as primary health information source regardless of their whereabouts or activities. This sentiment was captured by a participant when she described how her partner would contact her looking for reassurance about his fathering during her “away” time from primary caregiving responsibilities:

He uses social media in different ways… but not so much for parenting. He's like, ‘what do you think?’ and it’ll be me- he will send me videos and stuff when I’m out. Mostly to show me how horrible [my daughter] is for him. (participant laughs). I was figure skating last week and [my daughter] wouldn’t go down to sleep and he sent me a video of her crying. I’m like, ‘how is that helpful?’ (participant laughs). He's like, ‘what am I doing wrong?’ I’m like ‘you’re not doing anything wrong, you’re just not me.’” [T2]

As the aforementioned quote exemplifies, participants were held primarily accountable for the information needs of their family whether they were physically present or not.

### Attitudes regarding digital technologies for health information work

Participants’ characterization of their own and their partners’ apparent attitudes toward using digital technologies to seek health information followed similar gendered divisions as their direct use for health information work. Specifically, participants repeatedly indicated that while they were interested in actively engaging with digital technologies for health information work, they believed their partners were opposed to such activity. One participant shared a discussion she had with her partner in which he actively encouraged the participant to reduce the amount of digital health information seeking she conducted: “My husband's very- he's very like, ‘relax it’ll happen.’ Like ‘technology, put it away, don’t Google it.’ That's like- he said, ‘you Google everything.’”

Additionally, participants often described how their interest in using various forms of digital technologies to track health issues during the transition to parenting – such as their ovulation timing, maternal health, or fetal development – was not shared by their partners. For example, one participant described how her partner suggested she use digital technologies less by attributing her stress to her use of digital technology:

But he was- not that he didn’t take an interest, but he was the one who didn’t think it was necessary to do as much as I was doing. Like the, calendars and the timing and he's just like, oh, ‘you’re worrying too much,’ and he's like, ‘the stress is probably not good on any of us either.’ [T2]

Another participant described an instance when she found health information from online resources that suggested she may need to seek immediate medical attention, but was dismissed by her partner as needlessly stressing over information she encountered online:

I remember reading a lot about like, if you don’t feel your baby move like, you should do this and I would say that- that's what- like, I read that a lot and that's what- like I didn’t feel my son move one morning and I was like, ‘I’m going to the hospital.’ And like, again, my husband's like, ‘you’re on Google, you’re fine.’ But I wasn’t fine. And then I had him an hour later. So, I guess I think that that attributes to – like I obviously found that helped me.

Accounts such as these were common among this group of participants and epitomized a gendered dichotomy that existed between participants’ and their partners’ use of and attitudes toward digital technologies for health information work.

### Information curation: digital technologies encoding gender practices

As participants described the digital technologies that they and their partners used during the transition to parenting, it became apparent these resources may have been contributing to the gendered nature of the digital technology use these participants experienced through the reification of norms displayed in the aesthetics, form, and function of these platforms. For example, participants’ descriptions of fetal development tracking apps revealed how such resources were imbued with hegemonic feminine and masculine motifs. When such digital resources provided information that was curated toward mothers, participants indicated that the information itself was encoded as stereotypically feminine using colours and floral imagery, “it would say like, it has like, flowers when it's like you’re most fertile and then the pink flower was like your actual date of ovulation. So, that's what I used.” [T11] In contrast, participants described the hegemonic masculine imagery that was used for father-curated digital resources, and how such imagery reduced the perceived trustworthiness of the information these resources contained:

It was like a daddy app, so it was like relating it to like a size of a beer or something like that. It was totally like dad style… And then I think he would come to my pregnancy app to look at it if he wanted it to be a bit more serious” (T12)

In addition to the information curation process being encoded with gender norms, participants’ accounts of their and their male partners’ digital technology usage patterns suggested that such encoded gender norms extend to type and frequency of devices used during the transition to parenting. For example, several mothers shared with frustration that while their partners do engage with digital technologies, it is rarely related to health information work, “But he's not doing baby related stuff… No, it's football or fantasy crap, but anyway.” [T1] Other participants explained how the amount of time their partners spent socializing with friends while engaged with video games effected their family interactions:

He’ll come home, and he’ll be on his computer, on his little chat things, whatever, playing his games with people that he knows either online or actually in real life and he’ll spend a good portion of the night after she goes down playing and doing that. Sometimes he gets angry when she wakes up and he's gotta – ‘but I’m in a game!’ [T8]

While another participant described how her partner assumed it was acceptable to engage with digital technologies for work at home around their child, he did not participate in any digital health information work:

My husband does photography, so he does a lot of editing, but he does it through his phone and I find it frustrating because he does it when she's awake and I just wish he would do it later at night, so she isn’t seeing him staring at a phone all the time

I – we use them for different things. So, he didn’t engage as much in, like, the pregnancy in those types of apps or research or anything, but he plays online games and stuff like that. [T8]

For these participants, their partners’ active engagement with online sports, video games, and work, while frustrating, appeared to be accepted as normal and appropriate digital technology use behaviours for new fathers. Ultimately, such resignations to their partners’ digital technology use appeared to determine participants’ subsequent engagement with digital technologies, further encoding these normative gendered caregiving practices. For example, one participant described how her husband's ubiquitous use of technologies around their child caused her to engage with digital technologies less frequently:

I think I am more anti-technology because my husband like he comes home, and first thing he does, turn the TV on. So, if she's with daddy, I know that they’re just sitting there watching a program and it's usually Sponge Bob [cartoon] and then she's learning nothing from it. So, not his dependence, but his desire to have all of these things on, just pushes me to the total opposite. So, like when I’m pumping, I’m reading a book. [T2]

## Discussion

These findings provide important insight into the gendered nature of digital technology use during the transition to parenting. First, digital technologies appear to extend the gendered nature of health information work from the material to the digital realm and inequitably positioned woman/mothers as primary seekers of health information. Second, such gendered divisions of health work appear to be reinforced and normalized through digital resources that encode, and therefore reinscribe hegemonic gendered norms.

Gender identity is known to affect an individual's process of and attitudes toward health information seeking,^1,2^ and participants in this study provided insight into how the gendered aspect (and expectation) of health information seeking extends into digital spaces in multiple ways. These mothers frequently engaged in active *seeking*^3^ as they used digital technologies to purposefully look for health information for a range of health issues related to the transition to parenting. Due to the large amount of time spent engaged with digital resources, the experiences shared by these mothers also suggested they may engage in active *monitoring*^3^ – i.e., constantly scanning for health information related to the transition to parenting but not intentionally searching for a specific piece of information; however the majority of their experiences reflected active seeking.

Conversely, participants’ partners were described as engaging in proxy searching for health information by using them as an intermediary.^3,13^ During this process partners reportedly relied on the participants to send them personally curated health information, however they were often described to be disinterested in what the participants shared with them. Being relied on by their partners for digital health information effectively forced participants to embody the role of a lay information mediary^13^ as they would use digital technologies to search for, interpret, and share health information that they believed their partners could use. Furthermore, becoming a lay information mediary compelled these participants to actively engage in surveillance of their partners’ digital technology use to enable them to monitor what health information their partners were (not) reviewing. Adopting the role of lay information mediary places undue burden on mothers to conduct all the health information work for the family and highlights the role of digital technologies in maintaining and now extending into digitized realms, gendered inequities in the division of labour.^19^

Additionally, participants’ accounts of their and their partners’ attitudes toward digital technology use were embedded within sociocultural gender norms. Participants’ attitudes toward accessing as much health information as possible through a range of digital technologies resembled a monitoring approach to health information seeking;^1^ this monitoring attitude in new mothers has also been described elsewhere.^7,9,20^ Conversely, partners’ attitudes closely resembled a blunting approach to health information seeking as they were reportedly interested in the bare minimum amount of health information to address their needs,^21^ and at times relied exclusively on information provided to them at the direction of the infant's mothers. While in this study, digital technologies appeared to support the mothers’ monitoring behaviours by providing them with timely, readily available resources such as ovulation trackers and means to rapidly share/access information, simultaneously these technologies were oppressive and limiting of their autonomy.

Interestingly, digital technologies appeared to support their partners’ blunting behaviours by providing them with outlets for activities other than parenting, such as video gaming or paid work with which they could distract themselves from the health information tasks required for their family or childcare responsibilities more broadly. However, when participants were engaged in leisure or activities that took them away from the home, their partners used digital technologies to seek input, advice, or reassurance from participants – information often obtained by participants from the very time spent online that partners had problematized. While not fully explored in this study due to women compromising the entirety of the study sample, partners’ use of digital technologies to avoid health information may be an act of masculine gender performativity as acceptance of and seeking help or information for a health issue is often considered feminine behaviour.^12^ This gendered pattern of avoidance of self-health promotion via digital health information seeking appears to extend itself into men's roles as fathers. This insight builds on previous research regarding the gendered nature of caregiving more broadly to consider how inequities in childrearing responsibilities experienced by mothers have become augmented by digital technologies.

As participants described, the digital technologies they engaged with during the transition to parenting were overtly and covertly encoded with normative gender imagery. By encoding mother-focused resources with feminine imagery of delicate flowers and father-focused resources with masculine imagery of beer and sports, digital technology developers saliently establish who should and should not be interacting with their products. Furthermore, encoding digital technologies in a binary fashion inherently excludes any parent who does not identify with traditional sociocultural gender identities and heteronormative family growing practices. It also establishes the conditions in which new mothers are thrust into the role of lay information mediary, since fathers either (1) do not see themselves in the information resources, (2) do not believe the information contained in father-focused resources is as “serious” or trustworthy as the information contained in mother-focused resources, and/or (3) do not consider it part of their father role to engage in health information seeking in service of their family. Thus, the digital technologies that these mothers used to support their intense information needs during the transition to parenting may be an important social factor in maintaining inequitable division of health information work between new mothers and fathers. Furthermore, with the ever-evolving nature of online applications, platforms and devices coupled with the gendered nature of digital technology development^22^ it is conceivable that over time and left unchecked, future technologies could become even more oppressive and detrimental to the health and well-being of mothers positioned as curators and knowledge brokers of their families’ health information needs. For example, such technologies make new and expecting mothers, as well as their children, the subjects of digital surveillance as their activities, health concerns, and developmental milestones are catalogued by the digital technology developers.

To address the gendered division of health information labour digital technology developers should aim to establish high quality father-focused resources. Fathers often limit how readily they will engage in health information seeking since they often viewed it as the role of the mother.^11,12^ Therefore, creating father-focused digital technology resources may be a crucial step to ensuring the fathers see the resources as relevant to them by seeing overt gender cues embedded within them.^23^

Engaging fathers in meaningful health information seeking with such digital technologies will be a long process given the socially engrained nature of their health information avoidance. Therefore, parenting interest groups, health authorities, health policy makers, or public health officials who provide information resources for new parents could launch gender-transformative initiatives^24^ to promote fathers to become more engaged with health information. Such initiatives problematize and challenge gender inequities, such as fathers’ use of digital technologies to avoid health information work (e.g. playing video games), and empower groups to question and change their own behaviours. For example, several local “Dad Clubs” have formed within Ontario, Canada to offer father-only peer support and learning opportunities for new fathers. The goal of such groups is foster a positive outlook toward health information work among fathers, with the ultimate goal of reducing the amount of health information work that is required by partners of new mothers during the transition to parenting.

### Limitations

While this study provides important insight into the role of digital technologies in extending and reinforcing gendered health information work during the transition to parenting, it is not without its limitations. First, no males, fathers, or LGBTQ2 + individuals volunteered to participate in this study; thus these findings represent a specific gendered understanding of the transition to parenting as told by heterosexual, cis gendered women. Second, all participants indicated that they resided in urban areas, which limited how the influence of geography on participants’ use of digital technologies during the transition to parenting could be understood.

## Conclusion

This study has revealed that digital technologies tailored to new and expectant parents actively reinforced Western sociocultural heteronormative feminine and masculine gender roles regarding health information work. The participants in this study revealed that digital technologies facilitated their active health information seeking while simultaneously providing their partners with a means to absolve themselves from health information work related to the transition to parenting. This gendered dichotomy resulted in the participants becoming digital lay information mediaries for their partners. The content of the digital technologies designed for new and expectant parents appeared to reify this gendered division of health information labour, creating undue burden on new mothers to become the sole health information seeker and interpreter for the family, while normalizing their partners’ use of digital technologies for non-health-related purposes.

Further research should include rural populations to understand how rural factors (such as geographic isolation, limited internet connectivity, or traditional religious conservatism) may influence digital technology use during the transition to parenting. Further research should also purposefully target males, fathers, LGBTQ2+, and gender non-conforming groups to provide further insight into how parents of all gender identities and orientations use digital technologies during their transition to parenting. Finally, future studies could highlight how gender is involved in the surveillance structures that manifest through digital technology use by new and expecting parents’ during the transition to parenting. Understanding how parents of all backgrounds and identities engage with digital technologies for health information work can help inform the development of high-quality digital information resources designed for all types of parents. Doing so may promote fathers to engage more actively with health information and may ultimately reduce the inequitable health information work load currently carried by mothers.
